# Regulation of Mitochondrial Respiration by Hydrogen Sulfide

**DOI:** 10.3390/antiox12081644

**Published:** 2023-08-20

**Authors:** Dandan Huang, Guangqin Jing, Shuhua Zhu

**Affiliations:** College of Chemistry and Material Science, Shandong Agricultural University, Taian 271018, China; ddhuang@sdau.edu.cn (D.H.); jinggq@sdau.edu.cn (G.J.)

**Keywords:** hydrogen sulfide, mitochondria, oxidative phosphorylation, tricarboxylic acid cycle, mitochondrial respiratory complex

## Abstract

Hydrogen sulfide (H_2_S), the third gasotransmitter, has positive roles in animals and plants. Mitochondria are the source and the target of H_2_S and the regulatory hub in metabolism, stress, and disease. Mitochondrial bioenergetics is a vital process that produces ATP and provides energy to support the physiological and biochemical processes. H_2_S regulates mitochondrial bioenergetic functions and mitochondrial oxidative phosphorylation. The article summarizes the recent knowledge of the chemical and biological characteristics, the mitochondrial biosynthesis of H_2_S, and the regulatory effects of H_2_S on the tricarboxylic acid cycle and the mitochondrial respiratory chain complexes. The roles of H_2_S on the tricarboxylic acid cycle and mitochondrial respiratory complexes in mammals have been widely studied. The biological function of H_2_S is now a hot topic in plants. Mitochondria are also vital organelles regulating plant processes. The regulation of H_2_S in plant mitochondrial functions is gaining more and more attention. This paper mainly summarizes the current knowledge on the regulatory effects of H_2_S on the tricarboxylic acid cycle (TCA) and the mitochondrial respiratory chain. A study of the roles of H_2_S in mitochondrial respiration in plants to elucidate the botanical function of H_2_S in plants would be highly desirable.

## 1. Introduction

Hydrogen sulfide (H_2_S), as an environmental toxin, is now confirmed to be a biological mediator and plays essential roles in normal physiology and in the responses to different stresses [1,2]. H_2_S also regulates the responses to oxidative stress by interplaying with reactive oxygen species (ROS) at multiple levels [3,4] and protects mitochondrial function [5,6], maintaining mitochondrial homeostasis [7]. Mitochondria are the cells’ oxidation centers and power stations; they coordinate cell metabolism and immunity [8], and are both the source and the target of H_2_S. H_2_S can be produced inside or outside mitochondria, regulating mitochondrial energy metabolism, and maintaining mitochondrial functions under stress [3,9]. In mitochondria, the tricarboxylic acid (TCA) cycle is the final metabolic pathway of the three major nutrients (sugars, lipids, and amino acids) and the hub of the metabolism of sugars, lipids, and amino acids. The TCA cycle is a step in the process of respiration, after which high energy electrons are oxidized and ADPs are phosphorylated through the electron transport chain with the help of NADH, H^+^, and FADH_2_ to produce ATPs [10]. The mitochondrial respiratory chain, also called an electron transfer chain, is a continuous reaction system composed of a series of hydrogen transfer reactions and electron transfer reactions arranged in a specific order; it produces the majority of ROS, and supplies the cell with energy [11]. Respiratory chain complex I (NADH-ubiquinone oxidoreductase) oxidizes NADH, pumps protons from the inside of the mitochondrial inner membrane to the membrane gap, and transfers electrons to ubiquinone; complex II (succinate dehydrogenase) has a role in transferring electrons from succinic acid to ubiquinone; complex III (ubiquinone-cytochrome c oxidoreductase), an essential mitochondrial protein complex in the oxidative phosphorylation process, transfers electrons from ubiquinone to cytochrome c; complex IV (cytochrome *c* oxidase) pumps protons into the membrane gap and transfers electrons from cytochrome *c* to oxygen. These protons drive ATP synthesis by ATP synthase [12]. Disorder in the mitochondrial respiratory complexes is an essential cause of mitochondrial disease and aging [13]. In this paper, the articles about H_2_S, mitochondria, TCA cycle, and respiratory complexes were searched for, using Google Scholar, and about 20,000 results, dated after 2016, were obtained from databases and publishers, such as Web of Science, NCBI, Elsevier, Wiley, Springer, MDPI, et al. Based on these articles, the roles of H_2_S in regulating the process of mitochondrial respiration in recent years were reviewed.

## 2. Chemical and Biological Characteristics of H_2_S

H_2_S, as a colorless, corrosive gas, is poisonous and even lethal at high concentrations [14]; as a lipophilic molecule, it can diffuse readily through biological membranes. As a polar and hydrogen-bonding-capable molecule, H_2_S has a membrane permeability comparable to O_2_ and CO_2_, which are nonpolar. H_2_S can cross lipid bilayers with permeability coefficients from 0.5 cm/s to about 12 cm/s, which depend on the different membranes [15]. The solubility of H_2_S in pure water is up to 3.846 mg/g at 20 °C, and aqueous H_2_S is volatile due to its dissociation [16]. More than 80% of H_2_S in the water at physiological pH is dissociated to hydrosulfide anion (HS^−^) and then dissociated to sulfide anion (S^2−^) at a higher pH, and the rest of the H_2_S remains as an undissociated molecule [17]. H_2_S, as a weak diprotic acid, has pKa_1_ values of 6.98 at 25 °C and 6.76 at 37 °C [14]. Therefore, the availability of HS^−^ is high at neutral pH in vivo. The pKa values of the second dissociation are 17~19 at 25 °C [14]. Thus, alkaline sodium sulfide (Na_2_S) and sodium hydrosulfide (NaHS) solution can be applied as H_2_S sources to lower the pH.

The energies of orbitals for H_2_S (−10.47 eV) are lower than those of HS^−^ (−2.31 eV), indicating that the nucleophilicity of HS^−^ is higher than that of H_2_S [17]. With its negative charge and low electronegativity, HS^−^ can form a covalent bond with an electrophile (E^+^) by donating a pair of electrons, producing E-SH, and the product E-SH can also react with another E^+^ to form E-S-E [14]. This reactivity is the basis of the biological effect of H_2_S.

**Figure 1:**
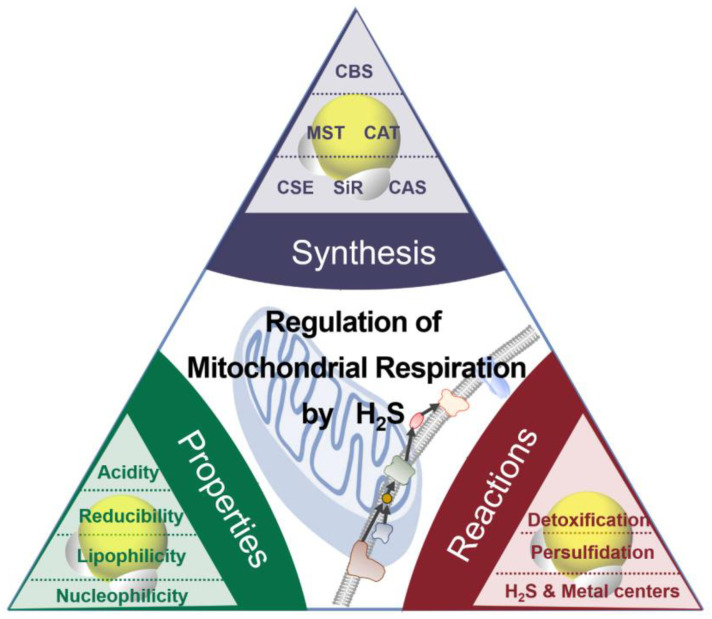
The synthesis, properties, and reaction of H_2_S.

The chemical properties of H_2_S (or HS^−^) as nucleophiles give the possibilities for two kinds of interaction between H_2_S and metals (Figure 1) [18]: (i) H_2_S (HS^−^) can bind noncovalently or coordinate the transitional metals as a ligand; (ii) H_2_S (HS^−^) can reduce the metal, accompanied by the production of HS^•^ and other downstream sulfur oxidation products. The positively charged transitional metal ions, such as iron and copper ions, can change valence by accepting an electron.

Cytochrome *c* oxidase (CcO), as a mitochondrial hemeprotein, contains copper centers, which are Cu_A_ and Cu_B_, and ferric heme a and a_3_ [19]. H_2_S can bind to and reduce CcO and serve as an electron donor. Ferric heme a_3_ can oxidize H_2_S with low concentrations to be HS^•^. The product HS^•^ is likely to react with HS^−^ to produce H_2_S_2_^•−^. Alternatively, HS^•^ can be oxidized by oxygen to form HSOO^•^. Despite inhibiting CcO, heme iron reduction promotes oxygen consumption, resulting in the stimulation of respiration [14,20]. At high levels, H_2_S binds to the O_2_-binding Cu_B_ center to be a Cu-SH complex that cannot be re-oxidized. Excessive H_2_S coordinates to ferric heme a_3_ forming an Fe-SH complex, eventually leading to irreversible inhibition of CcO. Cytochrome *c* has similar behavior in that heme ferric iron is reduced by H_2_S. Therefore, more reducing agents enter the electron transfer chain, consuming more oxygen. The inhibition of cytochrome *c* by H_2_S, to some extent, promotes CcO reduction and respiration [21].

H_2_S, a covalent hydride, is considered the simplest thiol, and its bond dissociation energy is about 385 kJ/mol, which is similar to that of the S-H bond in other thiols [22]. Both H_2_S and HS^−^ act as reductants. H_2_S can be oxidized by oxidants to substances with higher oxidation states, including sulfur (S^0^), sulfur dioxide (SO_2_), sulfite (SO_3_^2−^), sulfate (SO_4_^2−^), sulfur trioxide (SO_3_), and thiosulfate (S_2_O_3_^−^), and sulfonyl radical (HS^•^).

H_2_S can be oxidized by several biologically reactive species, such as nitrogen dioxide, hydroxyl radicals, peroxyl radicals, and superoxide radicals. The HS^•^ is the initial oxidation product of H_2_S. HS^•^ can be transformed into SO_2_^•−^ under the oxidation of O_2_, while O_2_ is catalyzed to be a superoxide radical (O_2_^•−^). O_2_^•−^ can be dismutated by superoxide dismutase into O_2_ and H_2_O_2_. The nucleophilic substitution of HS^−^ on H_2_O_2_ forms polysulfanes. Sulfenic acid (HSOH) formed by the reaction between HS^−^ and hydroperoxides (ROOH) can be transformed into HSSH by reacting with another HS^−^. The nucleophilic attack of HS^−^ on peroxynitrite (ONOOH) gives HSOH and NO_2_^−^. ^•^NO is involved in many vital physiological processes and signaling in mammals and plants, and has complex crosstalk with H_2_S signaling. H_2_S can reduce ^•^NO to form nitroxyl (HNO) or nitrososulfane (HSNO^•−^), and eventually leading to N_2_O and sulfane sulfur formation [23]. Oxidization of ^•^NO to NO_2_^−^ can also be facilitated by H_2_S. H_2_S can stimulate the formation of *S*-nitrosothiols (RSNO) of cysteine caused by ^•^NO.

In addition to *S*-nitrosothiols, persulfide (RSSH/RSS^−^) can be formed from the posttranslational modification of cysteines by H_2_S (HS^−^). H_2_S also reacts with GSSG to generate glutathione persulfide (GSSH) [24]. H_2_S can transfer sulfur with the catalysis of sulfide quinone oxidoreductase (SQR) to GSH to form GSSH [25].

Apart from their similar characteristics to thiols, disulfides, polysulfides, and hydroperoxides, persulfides attract increasing attention in biology as versatile molecules. Compared with thiols and H_2_S, persulfides are predicted to be more acidic and nucleophilic with a weaker S-H bond whose dissociation energy is 293 kJ/mol [26]. Thus, RSSH can reduce ferric cytochrome *c* to ferrous cytochrome *c* with the concomitant generation of RSS^•^. The cysteine residues modify the sulfur transferase (ST) structures involved in the H_2_S-producing process. The sulfur of the active site of the protein persulfides can be catalyzed by these enzymes to be thiols or sulfite. H_2_S can react with protein sulfenic acids (RSOH) to form persulfides (RSSO_2_H/RSSO_3_H) [14]. Iron-sulfur (Fe-S) clusters, as inorganic cofactors, especially bind to respiratory complexes, becoming involved in fundamental life processes such as energy production as well as electron transfer. The generation of persulfides (RSSH) involves Fe-S cluster synthesis, which is the crucial step of Fe-S assembly in mitochondria [27]. Persulfides are unstable in solution at room temperature and react with the outer and inner sulfur yielding sulfur and H_2_S (HS^−^) [28]. Persulfides/polysulfides contain sulfane sulfur, which has six valence electrons and no charge [29] and is mainly responsible for the biological activity attributed to H_2_S [30]. H_2_S is synthesized by the same enzymes involved in forming sulfane sulfur [31], suggesting a close relationship between H_2_S and sulfane sulfur and that these two reactive sulfur species always coexist [32,33]. It has been suggested that it is rather a sulfane sulfur, and not the H_2_S itself, that acts as a signaling molecule and is responsible for the biological actions of RSS. The term H_2_S is still used for narrative convenience in the following text.

Thus, H_2_S can signal through reduction and/or direct binding of metalloprotein heme centers, potent antioxidants through reactive oxygen species/reactive nitrogen species scavenging, and modifying proteins through persulfidation [5].

## 3. Enzymatic and Non-Enzymatic Biosynthesis of H_2_S

In mammals, homocysteine is catalyzed by cystathionine γ-lyase (CSE) to form H_2_S, α-ketobutyrate, and homolanthionine, or transforms to L-cysteine (L-Cys) through the transsulfuration pathway [34]. In mitochondria, L-Cys can be catalyzed by CSE to produce pyruvate and H_2_S, by cystathionine β-synthase (CBS) to form serine and H_2_S; CBS and CSE also involve transsulfuration and reverse transsulfuration pathways to regulate homocysteine metabolism [35]. Mitochondrial H_2_S can also be produced by cysteine aminotransferase (CAT) [36] and 3-mercaptopyruvate sulfurtransferase (3-MST) [31]. L-Cys is catalyzed by CAT to form 3-mercaptopyruvate which is then catalyzed by 3-MST to form pyruvate and H_2_S [7]. D-cysteine (D-Cys) can be catalyzed by D-amino acid oxidase in peroxisome to form 3-mercaptopyruvate, which is then catalyzed by 3-MST in mitochondria to form H_2_S. In addition, catalase, as a sulfide-sulfur oxidoreductase, catalyzes thioredoxin using NADPH to produce H_2_S in hypoxia in vitro (Figure 1) [37]. The non-enzymatic pathway also contributes to the content of endogenous H_2_S. Reactive sulfur species in persulfides, thiosulfate, and polysulfides can be reduced using NADP and NADPH into H_2_S [35].

Nevertheless, H_2_S may also impact the H_2_S-generating enzymes as a mediator. The glutathionylation of CBS (modified at Cys346) increases CBS activity [38]. Human CBS is also a hemeprotein. The binding between the ferrous heme in CBS and exogenous NO leads to the dissociation of Cys52 and His65 and the loss of CBS catalytic activity [39]. H_2_S reacts with ^•^NO and H_2_O_2_ to form nitrosothiol and polysulfanes, respectively, which may be promising to relieve the influence on CBS induced by reactive oxygen/nitrogen species. CSE can be activated when phosphorylated and oxidative stress also additionally induce CSE expression. CSE can undergo persulfidation, but the role of cysteine modification is still unknown [23]. High levels of H_2_S decrease CES expression or inhibit SP1 activation and CSE transcription [40].

In plants, the sources of H_2_S are more complicated than those in mammals. Plants can obtain H_2_S from the environment [41], sulfate assimilation [42], and endogenous generation [43,44]. The generation of endogenous H_2_S in plants also contains enzymatic and non-enzymatic pathways. These pathways are also included: the SiR pathway, in which sulfite reductase catalyzes sulfite to produce H_2_S; the CAS pathway, in which cyanoalanine synthase catalyzes L-Cys to produce cyanide and H_2_S; the L-/D-Cys pathway, in which L-/D-Cys is catalyzed by L-/D-cysteine desulfhydrase to form pyruvate and H_2_S; the cysteine synthase pathway, in which cysteine synthase catalyzes L-cysteine to form H_2_S [45,46]. In *Arabidopsis thaliana*, L-Cys and D-Cys are catalyzed by cysteine desulfhydrase to produce H_2_S; *O*-acetylserine (thiol) lyase (OAS-TL) also catalyzes cysteine to produce H_2_S in vitro [47]. The nitrogenase Fe-S cluster, like other classes of H_2_S synthase, is involved in the generation of H_2_S from L-Cys in mitochondria and plastid [48,49].

H_2_S exists widely in tissues. Improper levels of H_2_S cause harm, so it is crucial to manipulate H_2_S levels to maintain the beneficial effects of H_2_S [50]. The maintenance of mitochondrial sulfide homeostasis involving various enzymes is fundamental to ensure adequate energy production. Sulfide-consuming enzymes balance the sulfide level by catalyzing sulfide detoxification which can transfer sulfide to substances with higher oxidation states, e.g., persulfide, sulfite, sulfate, and thiosulfate. Excessive H_2_S in the mitochondria matrix is consumed by SQR to generate persulfide, and the electrons are released to ubiquinone and transferred to complex III. GSSH, formed from sulfide and GSH, is oxidized by persulfide dioxygenase (PDO, also referred to as ETHE1 in mitochondria) to generate sulfite that can be catalyzed by sulfite oxidase (SO) into sulfate. The sulfane sulfur is also transferred to sulfite and GSSH by thiosulfate sulfurtransferase (TST), forming thiosulfate [51,52]. OAS-TL contributes to sulfide consumption in the mitochondria of *Arabidopsis* and detoxifies sulfide to produce cysteine [53].

## 4. The regulation of Mitochondrial Function by H_2_S

Our previous paper [54] has preliminarily summarized the agents and methods used for H_2_S research and the progress of research on the regulation of H_2_S on plant metabolism and morphogenesis, abiotic stress tolerance, and the series of different post-translational modifications in which H_2_S is involved. It has been noted that regulation by H_2_S on mitochondrial function is a critical topic for the biological functions of H_2_S. H_2_S regulates mitochondrial oxidative stress by decreasing ROS content and enhancing the activities of the antioxidative enzymes in mitochondria, increases mitochondrial membrane fluidity and mitochondrial membrane potential [55], inhibits the opening of mitochondrial permeability transition pores [56], promotes mitochondrial biogenesis [57], protects against mitochondrial dysfunction [58,59], and regulates mitochondrial respiration [60]. The areas of regulation carried out by H_2_S on the mitochondrial processes are listed in Table 1. H_2_S has dual effects in regulating mitochondrial functions in mammals and plants. Generally, H_2_S exhibits its positive effects at low concentrations and toxic effects at high concentrations. The biological effects of H_2_S depend on its concentration and the different biological materials.

In mitochondria, the tricarboxylic acid (TCA) cycle oxidizes organic acids to release energy, and the mitochondrial electron transport chain synthesizes ATP by oxidative phosphorylation. H_2_S also impacts the TCA cycle and respiration in mitochondria.

### 4.1. The Regulation of the Tricarboxylic Acid (TCA) Cycle by H_2_S

The TCA cycle is the hub of energy metabolism inside mitochondria. Mitochondrial pyruvate dehydrogenase catalyzes the irreversible reaction that converts pyruvate into acetyl-CoA, which, together with oxaloacetate, is then catalyzed by citrate synthase to generate citrate. Citrate is converted by aconitase into isocitrate, which is then catalyzed by isocitrate dehydrogenase into α-ketoglutarate. With acetyl-CoA and NAD^+^, α-ketoglutarate is converted by α-ketoglutarate dehydrogenase into succinyl-CoA. Succinyl-CoA is catalyzed by succinyl-CoA synthetase to become succinate, coupling with the generation of GTP from GDP and Pi, which can be converted into ATP. Succinate dehydrogenase oxidizes succinate to generate fumarate. Fumarate is converted into malate by fumarase and further catalyzed by malate dehydrogenase into oxaloacetate that combines with another acetyl-CoA molecule to continue the TCA cycle [76].

In mammals, H_2_S regulates the TCA cycle to balance mitochondrial electron transport [77]. H_2_S increases lactate dehydrogenase activities [78] and promotes lactate accumulation by reducing the citrate synthase enzyme level of the TCA cycle [79]. A low concentration of GYY4137 (a slow-releasing H_2_S donor) enhances mitochondrial oxygen consumption, ATP production, and spare respiratory capacity, induces the *S*-sulfhydration of Cys163 in lactate dehydrogenase, and stimulates enzyme activity [80]. Under H_2_S stress, Belize fish increase cytochrome *c* oxidase and citrate synthase activities to tolerate higher levels of aquatic H_2_S without inhibiting mitochondrial oxygen consumption [81]. NaHS upregulates the activities of pyruvate dehydrogenase, malate dehydrogenase, isocitrate dehydrogenase, succinyl-CoA ligase, fumarate hydratase, succinate dehydrogenase of TCA cycle in db/db mice [82].

In plants, H_2_S can also regulate the TCA cycle in *Arabidopsis* via protein persulfidation [83]. H_2_S induces succinic dehydrogenase activity and promotes the efficiency of the TCA cycle in peach fruit against chilling injury [84]. H_2_S regulates the changes in the contents of citrate, aconitate, 2-oxoglutarate, fumarate, and oxaloacetate in *Malus hupehensis* Rehd. var. *pingyiensis* seedlings, recycles the TCA cycle to improve salt-stress recovery, and H_2_S overdose exaggerates salt-triggered metabolic perturbation [85]. H_2_S can modify the cysteine of enzymes to the persulfide involved in energy metabolism [9], and protein persulfidation is mainly involved in primary metabolic pathways, including the cycle [83]. Exogenous H_2_S inhibits isocitrate dehydrogenase activity by persulfidation and actives malic enzyme in peach fruit [86] and sweet pepper [87], suggesting that H_2_S mediates the TCA cycle in postharvest fruit responding to abiotic stress and the ripening process. However, excessive H_2_S inhibits the expression of pyruvate dehydrogenase complex, succinate dehydrogenase, and pyruvate kinase, reflecting energy dysfunction [88].

### 4.2. The Interplay of H_2_S and Mitochondrial Respiratory Complexes

Mitochondrial respiratory complex I (NADH: ubiquinone oxidoreductase) is a major contributor to the endogenous production of ROS, oxidized NADH from the TCA cycle in mitochondria, consisting of FMN molecules and Fe-S clusters [89]. *Yarrowia lipolytica* complex I has sulfur transferase subunit ST1 catalyzing the generation of H_2_S from 3-mercaptopyruvate, suggesting that complex I links with mitochondrial sulfur metabolism [52]. In rat liver mitochondria, 3-mercaptopyruvate at low concentrations stimulates mitochondrial electron transport; however, 3-mercaptopyruvate at high concentrations exhibits its inhibition [90]. Complex I in skeletal muscle is augmented, and the bioavailability and biosynthesis of H_2_S are suppressed in diabetic muscle; exogenous NaHS reduces the activity of complex I and improves H_2_S bioavailability [91]. Exogenous NaHS significantly increases the activity of complex I and restores it to normal levels [92]. Plant γ-carbonic anhydrase, a plausible source of H_2_S within plant leaves, encodes for a part of mitochondrial Complex I [93]. However, the interplay between H_2_S and complex I in plants is rarely reported.

Mitochondrial respiratory complex II (succinate: ubiquinone oxidoreductase), containing flavoprotein (Fp), iron-sulfur protein (Ip), CybL, and CybS, oxidizes succinate to become fumarate, and transfers electrons to ubiquinone, reduces the ubiquinone (Q) pool, contributing indirectly to the proton-motive force [94]. High sulfide oxidation flux can limit the pool of oxidized coenzyme Q (CoQ) accepting electrons from complexes I and II, potentially perturbing mitochondrial bioenergetics [95]. However, it has also been reported that NaHS has no significant effect on complex II in the cortex and hippocampus [92]. Mitochondrial sulfides: quinone oxidoreductase (SQR) catalyzes the sulfide oxidation pathway, transferring electrons to CoQ and coupling to complex III, which is critical against H_2_S poisoning [95]. CoQ deficiency causes the impairment of H_2_S oxidation, and CoQ supplementation regulates the levels of SQR, thiosulfate sulfurtransferase (TST), persulfide dioxygenase and sulfite oxidase (SO) in the H_2_S oxidation pathway, enhancing the free pool of CoQ to reduce oxidative stress [36]. There is a new redox cycle between SQOR and complex II at high H_2_S concentrations, reversing complex II and leading to the accumulation of succinate [96]. In plants, the activity of mitochondrial complex II is related to stomatal behavior [64]. H_2_S is involved in the feeding of electrons in complex II of mitochondria by quinone oxidoreductase [97] and modulates stomatal movement under abiotic stresses [98]. Mitochondrial complex II and SQR provide electrons and are involved in the biosynthesis of endogenous H_2_S under different conditions, and H_2_S triggers cell signaling activity and opens signal transduction pathways in plants [99].

Mitochondrial respiratory complex III (ubiquinol-cytochrome c reductase) transfers electrons from complex I or complex II-like enzymes to cytochrome *c* (Cyt C) [100,101]. H_2_S stimulates *Mycobacterium tuberculosis* respiration and bioenergetics predominantly via complex III [102], increases electron transport at complex III, and improves cellular metabolism against hyperglycemic injury [103]. The genes encoding ubiquinol-cytochrome c reductase of complex III in the mitochondrial ETC in leaves of poplar are upregulated by NaCl stress, exogenous cysteine accumulates H_2_S and regulates the expression of ubiquinol-cytochrome c reductase [104].

The oxidation of H_2_S can denote electrons directly to complex IV or indirectly via the initial reduction in Cyt C by sulfide. The mitochondrial sulfide oxidation pathway also connects to complex III. H_2_S reduces the Fe^3+^ of Cyt C to Fe^2+^, stimulates protein persulfidation, and indirectly transfers the electron to complex IV [21]. Mitochondrial respiratory complex IV (cytochrome *c* oxidase) contains heme copper and pumps protons across the inner mitochondrial membrane. High concentrations of H_2_S inhibit the binding of oxygen with complex IV, dissipate the inner mitochondrial membrane potential, and block aerobic ATP generation [25,105]. Excessive H_2_S inhibits mitochondrial complex IV and oxidative phosphorylation in Down syndrome [73] and increases superoxide dismutase activities leading to a decrease in ROS in cardiomyocytes under ischemia/reperfusion [106]. The intricate interplay between H_2_S, nitric oxide, carbon monoxide, and complex IV has been well-reviewed by Sarti and Arese [107]. H_2_S at toxic levels may inhibit cytochrome c oxidase activity and then inhibit ATP production under normoxic conditions, while in conjunction with hypoxia, H_2_S may promote the production of ATP under stress conditions [108]. H_2_S at high concentrations apparently inhibits the activity of mitochondrial complex IV and mitochondrial function [109]. Sulfite oxidase detoxifies sulfite in plant cells and relays electrons by heme b cofactor to cytochrome *c*, then to complex IV in the mitochondrial intermembrane space in humans [14]. AP39, an H_2_S donor, induces stomatal closure in a complex IV-dependent manner in *Arabidopsis thaliana* [64]. H_2_S at high concentration inhibits complex IV, and the inhibitory effect on complex IV contributes to the toxicity of H_2_S in plants [110,111].

Mitochondrial respiratory complex V (F_1_F_O_ ATPase) has eight different subunits, including two major subunits, F_O_ and F_1_ [112]. Complex V captures protons pumped by complexes I, III, and IV to produce ATP. Likewise, complex V synthesizes ATP with the electrochemical energy stored in its proton-motive force from complex II [113]. H_2_S increases the activity of complex V [92] and induces *S*-sulfhydration of the sulfhydryl groups of proteins yielding a hydropersulfide moiety (-SSH), which is critical for maintaining complex V activity in a physiological state, thereby supporting mitochondrial bioenergetics [77]. H_2_S affects the Ca^2+^-activated F_1_F_O_-ATPase activity but does not change the Mg^2+^-activated F_1_F_O_-ATPase activity in swine heart mitochondria [114]. Generally, low concentrations of H_2_S cause *S*-sulfhydration of complex V, increase the activity of complex V, and further enhance ATP generation [77]. However, H_2_S also induces oxidative stress, weakens the activity of ATPase, then leads to excessive mitochondrial fission [115]. Compared to animals, no precise results are reported on the meaning of H_2_S on F_1_F_O_ ATPase in plants.

**Figure 2:**
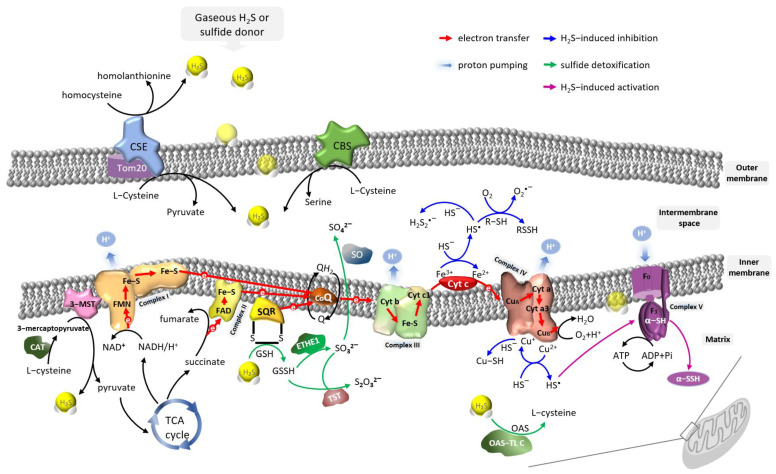
The possible pathways through which H_2_S regulates mitochondrial electron transport chain complexes.

The possible pathways through which H_2_S regulates the mitochondrial electron transport chain complexes are described in Figure 2.

Mitochondrial respiration is a vital process involving growth and development, disease occurrence and treatment in animals. The TCA cycle and mitochondrial respiratory complexes are also important for regulating disease occurrences and drug treatments. In animals, the dual effects of H_2_S and the proper dose of H_2_S have been confirmed in different biological processes, and the regulation by both exogenous and endogenous H_2_S of the TCA cycle and mitochondrial respiratory complexes are being studied extensively and deeply in different diseases. Differently from animals, plants, especially fruit, have different organs for people to utilize. Mitochondrial respiration also plays important roles in the development, maturation, ripening, and senescence of plants. The dual effects of H_2_S in plants have also been reported. However, current research on plant hydrogen sulfide focuses on plant growth and development and stress resistance, and the interplays between H_2_S and the TCA cycle and mitochondrial respiratory complexes are ignored to a certain extent by botanists. Although a few results show that H_2_S has regulatory effects on the critical enzymes in the TCA cycle and the complexes II, III, and IV in plant mitochondria, the results are still very preliminary, and the study of H_2_S effects on complex I and IV in plant mitochondria is still lacking. As a result, current research on regulating the TCA cycle and mitochondrial electron transport chain by H_2_S is still in its infancy and lags behind that in animals. Mitochondrial respiration is a vital process regulating fruit quality, especially the quality of the postharvest fruit. Finding out the effects of hydrogen sulfide, H_2_S, on the TCA cycle and mitochondrial respiratory complexes would help the study of H_2_S’s effects on plant biology.

## 5. Conclusions and Perspectives

The versatile chemical and biological characteristics of H_2_S ensure that it is a multifunctional bioactive small molecule. With mitochondria as its source and target, H_2_S modulates the mitochondrial energy metabolism by regulating the components of TCA and the electron transport chain via direct redox reaction or protein *S*-persulfidation. Although the regulation of the activities of these enzymes and the complexes by H_2_S are reported widely in the aspects of physiology and biochemistry, the structural mechanisms by which H_2_S reacts with these biomacromolecules remain unclear, such as the reactive sites of these biomacromolecules when reacting with H_2_S, the kinetics of these reactions, the factors that affect these reactions, and so on. Furthermore, little is understood about H_2_S and the modification caused by H_2_S regulating mitochondrial genes, such as *rps1* and *atp6*, involved in the critical processes of electron transport and ATP synthesis. The complication of obtaining intact mitochondrial electron transport chain complexes from living cells and the difference between in vitro and in vivo experiments also increase the difficulty of solving these issues. Knowledge of crystallography, molecular biology, and chemical biology is expected to be used to study the reaction between H_2_S and these enzymes and complexes more deeply and further explore the roles of H_2_S in regulating the mitochondrial respiratory chain and mitochondrial function. Compared with mammals, the roles of H_2_S in TCA and mitochondrial electron transport chain complexes in plants are poorly studied. Controlling respiration is vital for plants, especially for prolonging plant life under biotic and abiotic stress and the postharvest qualities of fruit and vegetables. H_2_S has been confirmed to exhibit excellent functions, maintaining the postharvest qualities of fruit and vegetables and enhancing the tolerance of plants to stresses. Elucidating the roles of H_2_S on TCA and the mitochondrial electron transport chain complexes in plants is also suggested to be essential work for the future to help understand the botanical function of H_2_S.

## Figures and Tables

**Table 1:** Mitochondrial processes affected by H_2_S.

Mitochondrial Processes	Biological Model	Usage of H_2_S	Biological Materials	References
Mitochondrial antioxidant system	suppresses ROS generation and increases the ratio of GSH/GSSG and levels of antioxidant enzymes, including SOD, GSH-Px, HO-1, and NQO-1	5 mg/kg NaHS	male Wistar rats	[61]
	inhibits ROS generation	80 μmol/kg NaHS	*db*/*db* mice	[60]
	reduces mitochondrial hydrogen peroxide accumulation	100 μM NaHS	cucumber seedling with cadmium stress	[62]
	enhances SOD, guaiacol peroxidase, and CAT activities in the mitochondria	0.05 mM NaHS	*Malus hupehensis* under NaCl stress	[55]
	enhances the capacity of the antioxidant system and reduces the accumulation of root mitochondrial ROS caused by waterlogging	200 μM NaHS	mangrove plant *Avicennia marina*	[63]
	increases cytosolic hydrogen peroxide levels and oxidation of the glutathione pool in GCs	100 nM AP39 (mitochondrial H_2_S donor)	*Arabidopsis*	[64]
	reduces H_2_O_2_ concentration, and keeps high activities of SOD, POD and CAT of mitochondria	0.05 mM NaHS	sweet cherry stigma and ovary	[65]
Mitochondrial membrane	hyperpolarizes mitochondrial inner potential	100 nM AP39	*Arabidopsis*	[64]
	decreases the mitochondrial permeability transition pores and increases mitochondrial membrane fluidity, mitochondrial membrane potential, and cytochrome c/a ratio	0.05 mM NaHS	*Malus hupehensis* under NaCl stress	[55]
	decreases mitochondrial membrane permeability, increases mitochondrial membrane fluidity, membrane potential, Cyt c/a	0.05 mM NaHS	sweet cherry stigma and ovary	[65]
Mitochondrial biogenesis	reduces ATP synthesis	10 μM esterase-triggered COS/H_2_S donor	BEAS 2B human lung epithelial cells	[66]
	decreases ATP production and restores the ratio of NAD^+^/NADH	80 μmol/kg NaHS	*db*/*db* mice	[60]
	increases cytosolic ATP	100 nM AP39	*Arabidopsis*	[64]
	increases the activities of cytochrome c oxidase, succinate dehydrogenase, H^+^-ATPase and Ca^2+^-ATPase	1.0 mM NaHS	Cucumber fruit	[67]
	increases H^+^-ATPase activity	0.05 mM NaHS	sweet cherry stigma and ovary	[65]
	increases the activities of succinate dehydrogenase, cytochrome c oxidase, H^+^-ATPase, and Ca^2+^-ATPase, maintains high ATP and ADP contents and energy level	0.4 mM NaHS	nectarine fruit	[68]
	enhances the activities of H^+^-ATPase, Ca^2+^-ATPase, cytochrome c oxidase, succinate dehydrogenase, maintains high energy status	0.5 mM NaHS	banana fruit	[69]
	maintains high energy charge, activates ATPases, cytochrome c oxidase, succinate dehydrogenase, glucokinase, fructokinase, glucose-6-phosphate dehydrogenase, and 6-phosphogluconate dehydrogenase	0.8 mM NaHS	broccoli	[70]
	increases ATPase activity and downregulates *CsVDAC* and *CsANT* expression	100 μM NaHS	cucumber seedling with cadmium stress	[62]
Mitochondrial function	enhances the expression and activity of sirtuin 3 and decreases mitochondrial acetylation levels in cardiomyocytes under hyperglycemia and hyperlipidemia	80 μmol/kg NaHS	*db*/*db* mice	[60]
	decreases the number of mitochondria and impairs mitochondrial function, induces severe apoptosis	5–40 μM NaHS	embryo-larval stages of zebrafish	[71]
	protects against root mitochondrial structure damage, maintains high mitochondrial potential, and alleviates root mitochondrial functional damage caused by waterlogging	200 μM NaHS	mangrove plant *Avicennia marina*	[63]
	inhibits the release of Cyt c from the mitochondria, reduces the opening of the mitochondrial permeability transition pore, and the activity of caspase-3-like protease	100 μM NaHS	cucumber (*Cucumis sativus* L) root tip cells	[72]
	maintains mitochondrial function	100 μM NaHS	cucumber seedling with cadmium stress	[62]
Mitochondrial respiration	decreases mitochondrial respiratory rate	80 μmol/kg NaHS	*db*/*db* mice	[60]
	inhibits mitochondrial complex IV and suppresses oxidative phosphorylation in Down syndrome	CBS-derived H_2_S	female dermal fibroblasts	[73]
	upregulates the alternative respiratory pathway	200 μM NaHS	mangrove plant *Avicennia marina*	[63]
	reduces the acetylation of ATP synthase mitochondrial F1 complex assembly factor 1	80 μmol/kg NaHS	*db*/*db* mice	[60]
	represses the TCA pathway, induces genes encoding mitochondrial respiratory chain complexes I, II, and III	0.7 mM NaHS	fresh-cut apple	[74]
	activates AOX-mediated cyanide-resistant respiration pathway	12 μM NaHS	*Arabidopsis* seeds	[75]
